# Supplementary material for: Biotechnological Potential of the Stress Response and Plant Cell Death Regulators Proteins in the Biofuel Industry
Source: Cells. 2023 Aug 8;12(16):2018. doi: 10.3390/cells12162018 (PMC10453534; doi:10.3390/cells12162018)
Supplement: Supplementary file 1 [file cells-12-02018-s001.zip › Additional file 1.pdf]

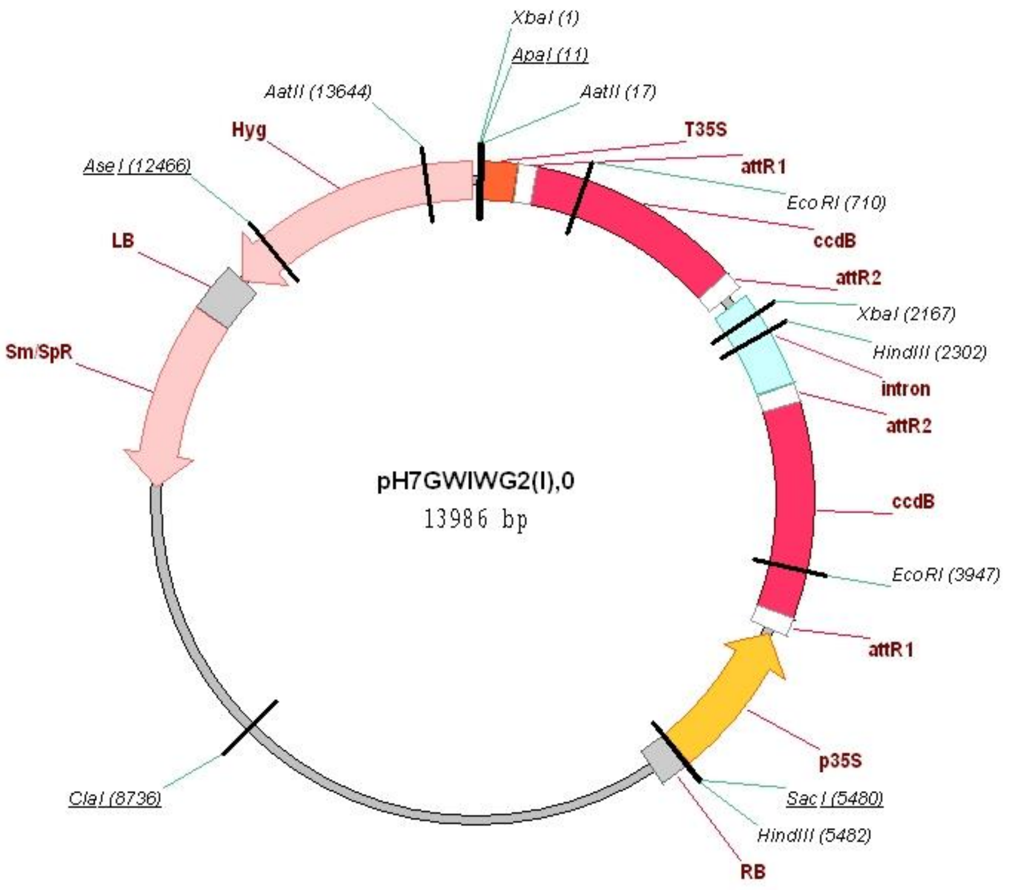

ID pH7GWIWG2(I),0 standard; circular DNA; ; 13986 BP.

CC This file is created by Vector NTI

CC http://www.informaxinc.com/

CC VNTDATE|342016955|

CC VNTDBDATE|342018061|

CC LSOWNER|

CC VNTAUTHORNAME|Mansour Karimi|

CC VNTAUTHORTEL|+3193313850|

CC VNTAUTHOREML|mansour.karimi@psb.ugent.be|

FH Key Location/Qualifiers

FH

FT intron complement(2035..2678)

```
FT                /vntifkey="15"
FT                /label=intron
FT   misc_recomb     complement(4273..4397)
FT                /vntifkey="86"
FT                /label=attR1
FT   misc_recomb     complement(2693..2817)
FT                /vntifkey="86"
FT                /label=attR2
FT   misc_feature    complement(2818..4272)
FT                /vntifkey="21"
FT                /label=CmR-ccdB
FT   terminator      28..253
FT                /vntifkey="43"
FT                /label=T35S
FT   misc_recomb     263..387
FT                /vntifkey="86"
FT                /label=attR1
FT   misc_recomb     1843..1967
FT                /vntifkey="86"
FT                /label=attR2
FT   misc_feature    388..1842
FT                /vntifkey="21"
FT                /label=CmR-ccdB
FT   misc_signal     5482..5681
FT                /vntifkey="87"
FT                /label=RB
```

FT misc_structure complement(10585..11834)

FT /vntifkey="88"

FT /label=Sm/SpR

FT misc_signal 11840..12172

FT /vntifkey="87"

FT /label=LB

FT misc_marker complement(12176..13962)

FT /vntifkey="22"

FT /label=Hyg

FT promoter complement(4425..5462)

FT /vntifkey="29"

FT /label=p35S

SQ Sequence 13986 BP; 3509 A; 3559 C; 3558 G; 3360 t;

ctagagggcc cgacgtcgca tgcctgcagg tcactggatt ttggttttag gaattagaaa 60

ttttattgat agaagtattt tacaaataca aatacatact aagggtttct tatatgctca 120

acacatgagc gaaaccctat aagaacccta attcccttat ctgggaacta ctcacacatt 180

attctggaga aaaatagaga gagatagatt tgtagagaga gactggtgat ttttgcggac 240

tctagcatgg ccgcgggata tcacaagttt gtacaaaaaa gctgaacgag aaacgtaaaa 300

tgatataaat atcaatatat taaattagat tttgcataaa aaacagacta cataatactg 360

taaaacacaa catatccagt cactatggcg gccgcattag gcaccccagg ctttacactt 420

tatgcttccg gctcgtataa tgtgtggatt ttgagttagg atccggcgag attttcagga 480

gctaaggaag ctaaaatgga gaaaaaaatc actggatata ccaccgttga tatatcccaa 540

tggcatcgta aagaacattt tgaggcattt cagtcagttg ctcaatgtac ctataaccag 600

accgttcagc tggatattac ggccttttta aagaccgtaa agaaaaataa gcacaagttt 660

tatccggcct ttattcacat tcttgcccgc ctgatgaatg ctcatccgga attccgtatg 720

gcaatgaaag acggtgagct ggtgatatgg gatagtgttc acccttgtta caccgttttc 780

catgagcaaa ctgaaacgtt ttcatcgctc tggagtgaat accacgacga tttccggcag 840

tttctacaca tatattcgca agatgtggcg tgttacggtg aaaacctggc ctatttccct 900

aaagggttta ttgagaatat gtttttcgtc tcagccaatc cctgggtgag tttcaccagt 960

tttgatttaa acgtggccaa tatggacaac ttcttcgccc ccgttttcac catgggcaaa 1020

tattatacgc aaggcgacaa ggtgctgatg ccgctggcga ttcaggttca tcatgccgtc 1080

tgtgatggct tccatgtcgg cagaatgctt aatgaattac aacagtactg cgatgagtgg 1140

cagggcgggg cgtaaacgcg tggatccggc ttactaaaag ccagataaca gtatgcgtat 1200

ttgcgcgctg atttttgcgg tataagaata tatactgata tgtatacccg aagtatgtca 1260

aaaagaggtg tgctatgaag cagcgtatta cagtgacagt tgacagcgac agctatcagt 1320

tgctcaaggc atatatgatg tcaatatctc cggtctggta agcacaacca tgcagaatga 1380

agcccgtcgt ctgcgtgccg aacgctggaa agcggaaaat caggaaggga tggctgaggt 1440

cgcccggttt attgaaatga acggctcttt tgctgacgag aacagggact ggtgaaatgc 1500

agtttaaggt ttacacctat aaaagagaga gccgttatcg tctgtttgtg gatgtacaga 1560

gtgatattat tgacacgccc gggcgacgga tggtgatccc cctggccagt gcacgtctgc 1620

tgtcagataa agtctcccgt gaactttacc cggtggtgca tatcggggat gaaagctggc 1680

gcatgatgac caccgatatg gccagtgtgc cggtctccgt tatcggggaa gaagtggctg 1740

atctcagcca ccgcgaaaat gacatcaaaa acgccattaa cctgatgttc tggggaatat 1800

aaatgtcagg ctcccttata cacagccagt ctgcaggtcg accatagtga ctggatatgt 1860

tgtgttttac agtattatgt agtctgtttt ttatgcaaaa tctaatttaa tatattgata 1920

tttatatcat tttacgtttc tcgttcagct ttcttgtaca aagtggtgat atcactagtg 1980

cggccgcctg caggtcgacc atatggtcga cctgcaggcg gccgcactag tgatgctgtt 2040

atgttcagtg tcaagctgac ctgcaaacac gttaaatgct aagaagttag aatatatgag 2100

acacgttaac tggtatatga ataagctgta aataaccgag tataaactca ttaactaata 2160

tcacctctag agtataatat aatcaaattc gacaatttga ctttcaagag taggctaatg 2220

taaaatcttt atatatttct acaatgttca aagaaacagt tgcatctaaa cccctatggc 2280

catcaaattc aatgaacgct aagcttaata tgactctcaa taaagtctca taccaacaag 2340

tgccacctta ttcaaccatc aagaaaaaag ccaaaattta tgctactcta aggaaaactt 2400

cactaaagaa gacgatttag agtgttttac caagaatttc tgtcatctta ctaaacaact 2460

aaagatcggt gtgatacaaa acctaatctc attaaagttt atgctaaaat aagcataatt 2520

ttacccacta agcgtgacca gataaacata actcagcaca ccagagcata tatattggtg 2580

gctcaaatca tagaaactta cagtgaagac acagaaagcc gtaagaagag gcaagagtat 2640

gaaaccttac ctcatcattt ccatgaggtt gcttctgatc ccgcgggata tcaccacttt 2700

gtacaagaaa gctgaacgag aaacgtaaaa tgatataaat atcaatatat taaattagat 2760

tttgcataaa aaacagacta cataatactg taaaacacaa catatccagt cactatggtc 2820

gacctgcaga ctggctgtgt ataagggagc ctgacattta tattccccag aacatcaggt 2880

taatggcgtt tttgatgtca ttttcgcggt ggctgagatc agccacttct tccccgataa 2940

cggagaccgg cacactggcc atatcggtgg tcatcatgcg ccagctttca tccccgatat 3000

gcaccaccgg gtaaagttca cgggagactt tatctgacag cagacgtgca ctggccaggg 3060

ggatcaccat ccgtcgcccg ggcgtgtcaa taatatcact ctgtacatcc acaaacagac 3120

gataacggct ctctctttta taggtgtaaa ccttaaactg catttcacca gtccctgttc 3180

tcgtcagcaa aagagccgtt catttcaata aaccgggcga cctcagccat cccttcctga 3240

ttttccgctt tccagcgttc ggcacgcaga cgacgggctt cattctgcat ggttgtgctt 3300

accagaccgg agatattgac atcatatatg ccttgagcaa ctgatagctg tcgctgtcaa 3360

ctgtcactgt aatacgctgc ttcatagcac acctcttttt gacatacttc gggtatacat 3420

atcagtatat attcttatac cgcaaaaatc agcgcgcaaa tacgcatact gttatctggc 3480

ttttagtaag ccggatccac gcgtttacgc cccgccctgc cactcatcgc agtactgttg 3540

taattcatta agcattctgc cgacatggaa gccatcacag acggcatgat gaacctgaat 3600

cgccagcggc atcagcacct tgtcgccttg cgtataatat ttgcccatgg tgaaaacggg 3660

ggcgaagaag ttgtccatat tggccacgtt taaatcaaaa ctggtgaaac tcacccaggg 3720

attggctgag acgaaaaaca tattctcaat aaacccttta gggaaatagg ccaggttttc 3780

accgtaacac gccacatctt gcgaatatat gtgtagaaac tgccggaaat cgtcgtggta 3840

ttcactccag agcgatgaaa acgtttcagt ttgctcatgg aaaacggtgt aacaagggtg 3900

aacactatcc catatcacca gctcaccgtc tttcattgcc atacggaatt ccggatgagc 3960

attcatcagg cgggcaagaa tgtgaataaa ggccggataa aacttgtgct tatttttctt 4020

tacggtcttt aaaaaggccg taatatccag ctgaacggtc tggttatagg tacattgagc 4080

aactgactga aatgcctcaa aatgttcttt acgatgccat tgggatatat caacggtggt 4140

atatccagtg attttttct ccattttagc ttccttagct cctgaaaatc tcgccggatc 4200

ctaactcaaa atccacacat tatacgagcc ggaagcataa agtgtaaagc ctggggtgcc 4260

taatgcggcc gccatagtga ctggatatgt tgtgttttac agtattatgt agtctgtttt 4320

ttatgcaaaa tctaatttaa tatattgata tttatatcat tttacgtttc tcgttcagct 4380

tttttgtaca aacttgtgat atcactagtg cggccgcctg caggtcgact agaatagtaa 4440

attgtaatgt tgtttgttgt ttgttttgtt gtggtaattg ttgtaaaaat acggatcgtc 4500

ctgcagtcct ctccaaatga aatgaacttc cttatataga ggaagggtct tgcgaaggat 4560

agtgggattg tgcgtcatcc cttacgtcag tggagatatc acatcaatcc acttgctttg 4620

aagacgtggt tggaacgtct tctttttcca cgatgctcct cgtgggtggg ggtccatctt 4680

tgggaccact gtcggcagag gcatcttgaa cgatagcctt tcctttatcg caatgatggc 4740

atttgtaggt gccaccttcc ttttctactg tccttttgat gaagtgacag atagctgggc 4800

aatggaatcc gaggaggttt cccgatatta ccctttgttg aaaagtctca atagcccttt 4860

ggtcttctga gactgtatct ttgatattct tggagtagac gagagtgtcg tgctccacca 4920

tgttgacgaa gattttcttc ttgtcattga gtcgtaaaag actctgtatg aactgttcgc 4980

cagtcttcac ggcgagttct gttagatcct cgatctgaat ttttgactcc atggcctttg 5040

attcagtagg aactactttc ttagagactc caatctctat tacttgcctt ggtttatgaa 5100

gcaagccttg aatcgtccat actggaatag tacttctgat cttgagaaat atatctttct 5160

ctgtgttctt gatgcagtta gtcctgaatc ttttgactgc atctttaacc ttcttgggaa 5220

ggtatttgat ctcctggaga ttattactcg ggtagatcgt cttgatgaga cctgccgcgt 5280

aggcctctct aaccatctgt gggtcagcat tctttctgaa attgaagagg ctaatcttct 5340

cattatcggt ggtgaacatg gtatcgtcac cttctccgtc gaactttctt cctagatcgt 5400

agagatagag aaagtcgtcc atggtgatct ccggggcaaa ggagatcagc ttggctctag 5460

tcgaccatat gggagagctc aagcttagct tgagcttgga tcagattgtc gtttcccgcc 5520

ttcagtttaa actatcagtg tttgacagga tatattggcg ggtaaaccta agagaaaaga 5580

gcgtttatta gaataacgga tatttaaaag ggcgtgaaaa ggtttatccg ttcgtccatt 5640

tgtatgtgca tgccaaccac agggttcccc tcgggatcaa agtactttga tccaacccct 5700

ccgctgctat agtgcagtcg gcttctgacg ttcagtgcag ccgtcttctg aaaacgacat 5760

gtcgcacaag tcctaagtta cgcgacaggc tgccgccctg cccttttcct ggcgttttct 5820

tgtcgcgtgt tttagtcgca taaagtagaa tacttgcgac tagaaccgga gacattacgc 5880

catgaacaag agcgccgccg ctggcctgct gggctatgcc cgcgtcagca ccgacgacca 5940

ggacttgacc aaccaacggg ccgaactgca cgcggccggc tgcaccaagc tgttttccga 6000

gaagatcacc ggcaccaggc gcgaccgccc ggagctggcc aggatgcttg accacctacg 6060

ccctggcgac gttgtgacag tgaccaggct agaccgcctg gcccgcagca cccgcgacct 6120

actggacatt gccgagcgca tccaggaggc cggcgcgggc ctgcgtagcc tggcagagcc 6180

gtgggccgac accaccacgc cggccggccg catggtgttg accgtgttcg ccggcattgc 6240

cgagttcgag cgttccctaa tcatcgaccg cacccggagc gggcgcgagg ccgccaaggc 6300

ccgaggcgtg aagtttggcc cccgccctac cctcaccccg gcacagatcg cgcacgcccg 6360

cgagctgatc gaccaggaag gccgcaccgt gaaagaggcg gctgcactgc ttggcgtgca 6420

tcgctcgacc ctgtaccgcg cacttgagcg cagcgaggaa gtgacgccca ccgaggccag 6480

gcggcgcggt gccttccgtg aggacgcatt gaccgaggcc gacgccctgg cggccgccga 6540

gaatgaacgc caagaggaac aagcatgaaa ccgcaccagg acggccagga cgaaccgttt 6600

ttcattaccg aagagatcga ggcggagatg atcgcggccg ggtacgtgtt cgagccgccc 6660

gcgcacgtct caaccgtgcg gctgcatgaa atcctggccg gtttgtctga tgccaagctg 6720

gcggcctggc cggccagctt ggccgctgaa gaaaccgagc gccgccgtct aaaaaggtga 6780

tgtgtatttg agtaaaacag cttgcgtcat gcggtcgctg cgtatatgat gcgatgagta 6840

aataaacaaa tacgcaaggg gaacgcatga aggttatcgc tgtacttaac cagaaaggcg 6900

ggtcaggcaa gacgaccatc gcaacccatc tagcccgcgc cctgcaactc gccggggccg 6960

atgttctgtt agtcgattcc gatccccagg gcagtgcccg cgattgggcg gccgtgcggg 7020

aagatcaacc gctaaccgtt gtcggcatcg accgcccgac gattgaccgc gacgtgaagg 7080

ccatcggccg gcgcgacttc gtagtgatcg acggagcgcc ccaggcggcg gacttggctg 7140

tgtccgcgat caaggcagcc gacttcgtgc tgattccggt gcagccaagc ccttacgaca 7200

tatgggccac cgccgacctg gtggagctgg ttaagcagcg cattgaggtc acggatggaa 7260

ggctacaagc ggcctttgtc gtgtcgcggg cgatcaaagg cacgcgcatc ggcggtgagg 7320

ttgccgaggc gctggccggg tacgagctgc ccattcttga gtcccgtatc acgcagcgcg 7380

tgagctaccc aggcactgcc gccgccggca caaccgttct tgaatcagaa cccgagggcg 7440

acgctgcccg cgaggtccag gcgctggccg ctgaaattaa atcaaaactc atttgagtta 7500

atgaggtaaa gagaaaatga gcaaaagcac aaacacgcta agtgccggcc gtccgagcgc 7560

acgcagcagc aaggctgcaa cgttggccag cctggcagac acgccagcca tgaagcgggt 7620

caactttcag ttgccggcgg aggatcacac caagctgaag atgtacgcgg tacgccaagg 7680

caagaccatt accgagctgc tatctgaata catcgcgcag ctaccagagt aaatgagcaa 7740

atgaataaat gagtagatga attttagcgg ctaaaggagg cggcatggaa aatcaagaac 7800

aaccaggcac cgacgccgtg gaatgcccca tgtgtggagg aacgggcggt tggccaggcg 7860

taagcggctg ggttgtctgc cggccctgca atggcactgg aacccccaag cccgaggaat 7920

cggcgtgacg gtcgcaaacc atccggcccg gtacaaatcg gcgcggcgct gggtgatgac 7980

ctggtggaga agttgaaggc cgcgcaggcc gcccagcggc aacgcatcga ggcagaagca 8040

cgccccggtg aatcgtggca agcggccgct gatcgaatcc gcaaagaatc ccggcaaccg 8100

ccggcagccg gtgcgccgtc gattaggaag ccgcccaagg gcgacgagca accagatttt 8160

ttcgttccga tgctctatga cgtgggcacc cgcgatagtc gcagcatcat ggacgtggcc 8220

gttttccgtc tgtcgaagcg tgaccgacga gctggcgagg tgatccgcta cgagcttcca 8280

gacgggcacg tagaggtttc cgcagggccg gccggcatgg ccagtgtgtg ggattacgac 8340

ctggtactga tggcggtttc ccatctaacc gaatccatga accgataccg ggaagggaag 8400

ggagacaagc ccggccgcgt gttccgtcca cacgttgcgg acgtactcaa gttctgccgg 8460

cgagccgatg gcggaaagca gaaagacgac ctggtagaaa cctgcattcg gttaaacacc 8520

acgcacgttg ccatgcagcg tacgaagaag gccaagaacg gccgcctggt gacggtatcc 8580

gaggggtgaag ccttgattag ccgctacaag atcgtaaaga gcgaaaccgg gcggccggag 8640

tacatcgaga tcgagctagc tgattggatg taccgcgaga tcacagaagg caagaacccg 8700

gacgtgctga cggttcaccc cgattacttt ttgatcgatc ccggcatcgg ccgttttctc 8760

taccgcctgg cacgccgcgc cgcaggcaag gcagaagcca gatggttgtt caagacgatc 8820

tacgaacgca gtggcagcgc cggagagttc aagaagttct gtttcaccgt gcgcaagctg 8880

atcgggtcaa atgacctgcc ggagtacgat ttgaaggagg aggcggggca ggctggcccg 8940

atcctagtca tgcgctaccg caacctgatc gagggcgaag catccgccgg ttcctaatgt 9000

acggagcaga tgctagggca aattgcccta gcaggggaaa aaggtcgaaa aggtctcttt 9060

cctgtggata gcacgtacat tgggaaccca aagccgtaca ttgggaaccg gaacccgtac 9120

attgggaacc caaagccgta cattgggaac cggtcacaca tgtaagtgac tgatataaaa 9180

gagaaaaaag gcgatttttc cgcctaaaac tctttaaaac ttattaaaac tcttaaaacc 9240

cgcctggcct gtgcataact gtctggccag cgcacagccg aagagctgca aaaagcgcct 9300

acccttcggt cgctgcgctc cctacgcccc gccgcttcgc gtcggcctat cgcggccgct 9360

ggccgctcaa aaatggctgg cctacggcca ggcaatctac cagggcgcgg acaagccgcg 9420

ccgtcgccac tcgaccgccg gcgcccacat caaggcaccc tgcctcgcgc gtttcggtga 9480

tgacggtgaa aacctctgac acatgcagct cccggagacg gtcacagctt gtctgtaagc 9540

ggatgccggg agcagacaag cccgtcaggg cgcgtcagcg ggtgttggcg ggtgtcgggg 9600

cgcagccatg acccagtcac gtagcgatag cggagtgtat actggcttaa ctatgcggca 9660

tcagagcaga ttgtactgag agtgcaccat atgcggtgtg aaataccgca cagatgcgta 9720

aggagaaaat accgcatcag gcgctcttcc gcttcctcgc tcactgactc gctgcgctcg 9780

gtcgttcggc tgcggcgagc ggtatcagct cactcaaagg cggtaatacg gttatccaca 9840

gaatcagggg ataacgcagg aaagaacatg tgagcaaaag gccagcaaaa ggccaggaac 9900

cgtaaaaagg ccgcgttgct ggcgtttttc cataggctcc gcccccctga cgagcatcac 9960

aaaaatcgac gctcaagtca gaggtggcga aacccgacag gactataaag ataccaggcg 10020

tttccccctg gaagctccct cgtgcgctct cctgttccga ccctgccgct taccggatac 10080

ctgtccgcct ttctcccttc gggaagcgtg gcgctttctc atagctcacg ctgtaggtat 10140

ctcagttcgg tgtaggtcgt tcgctccaag ctgggctgtg tgcacgaacc ccccgttcag 10200

cccgaccgct gcgccttatc cggtaactat cgtcttgagt ccaacccggt aagacacgac 10260

ttatcgccac tggcagcagc cactggtaac aggattagca gagcgaggta tgtaggcggt 10320

gctacagagt tcttgaagtg gtggcctaac tacggctaca ctagaaggac agtatttggt 10380

atctgcgctc tgctgaagcc agttaccttc ggaaaaagag ttggtagctc ttgatccggc 10440

aaacaaacca ccgctggtag cggtggtttt tttgtttgca agcagcagat tacgcgcaga 10500

aaaaaaggat ctcaagaaga tcctttgatc ttttctacgg ggtctgacgc tcagtggaac 10560

gaaaactcac gttaagggat tttggtcatg catgatatat ctcccaattt gtgtagggct 10620

tattatgcac gcttaaaaat aataaaagca gacttgacct gatagtttgg ctgtgagcaa 10680

ttatgtgctt agtgcatcta atcgcttgag ttaacgccgg cgaagcggcg tcggcttgaa 10740

cgaatttcta gctagacatt atttgccgac taccttggtg atctcgcctt tcacgtagtg 10800

gacaaattct tccaactgat ctgcgcgcga ggccaagcga tcttcttctt gtccaagata 10860

agcctgtcta gcttcaagta tgacgggctg atactgggcc ggcaggcgct ccattgccca 10920

gtcggcagcg acatccttcg gcgcgatttt gccggttact gcgctgtacc aaatgcggga 10980

caacgtaagc actacatttc gctcatcgcc agcccagtcg ggcggcgagt tccatagcgt 11040

taaggtttca tttagcgcct caaatagatc ctgttcagga accggatcaa agagttcctc 11100

cgccgctgga cctaccaagg caacgctatg ttctcttgct tttgtcagca agatagccag 11160

atcaatgtcg atcgtggctg gctcgaagat acctgcaaga atgtcattgc gctgccattc 11220

tccaaattgc agttcgcgct tagctggata acgccacgga atgatgtcgt cgtgcacaac 11280

aatggtgact tctacagcgc ggagaatctc gctctctcca ggggaagccg aagtttccaa 11340

aaggtcgttg atcaaagctc gccgcgttgt ttcatcaagc cttacggtca ccgtaaccag 11400

caaatcaata tcactgtgtg gcttcaggcc gccatccact gcggagccgt acaaatgtac 11460

ggccagcaac gtcggttcga gatggcgctc gatgacgcca actacctctg atagttgagt 11520

cgatacttcg gcgatcaccg cttccccat gatgtttaac tttgttttag ggcgactgcc 11580

ctgctgcgta acatcgttgc tgctccataa catcaaacat cgacccacgg cgtaacgcgc 11640

ttgctgcttg gatgcccgag gcatagactg taccccaaaa aaacatgtca taacaagaag 11700

ccatgaaaac cgccactgcg ccgttaccac cgctgcgttc ggtcaaggtt ctggaccagt 11760

tgcgtgacgg cagttacgct acttgcatta cagcttacga accgaacgag gcttatgtcc 11820

actgggttcg tgcccgaatt gatcacaggc agcaacgctc tgtcatcgtt acaatcaaca 11880

tgctaccctc cgcgagatca tccgtgtttc aaacccggca gcttagttgc cgttcttccg 11940

aatagcatcg gtaacatgag caaagtctgc cgccttacaa cggctctccc gctgacgccg 12000

tcccggactg atgggctgcc tgtatcgagt ggtgattttg tgccgagctg ccggtcgggg 12060

agctgttggc tggctggtgg caggatatat tgtggtgtaa acaaattgac gcttagacaa 12120

cttaataaca cattgcggac gtttttaatg tactgaatta acgccgaatt gaattatcag 12180

cttgcatgcc ggtcgatcta gtaacataga tgacaccgcg cgcgataatt tatcctagtt 12240

tgcgcgctat attttgtttt ctatcgcgta ttaaatgtat aattgcggga ctctaatcat 12300

aaaaacccat ctcataaata acgtcatgca ttacatgtta attattacat gcttaacgta 12360

attcaacaga aattatatga taatcatcgc aagaccggca acaggattca atcttaagaa 12420

actttattgc caaatgtttg aacgatctgc ttgactctag ggaattaatt cctgaatcac 12480

tgcgaccggc cctcccgcga cccagccgag cgagcttagc gaactgtgga cgagaactgt 12540

gccaccaagc gtaaggccgt tctctcgcat ttgccttgct aggctcgcgc gagttgctgg 12600

ctgaggcgtt ctcgaaatca gctcttgttc ggtcggcatc tactctattc ctttgccctc 12660

ggacgagtgc tggggcgtcg gtttccacta tcggcgagta cttctacaca gccatcggtc 12720

cagacggccg cgcttctgcg ggcgatttgt gtacgcccga cagtcccggc tccggatcgg 12780

acgattgcgt cgcatcgacc ctgcgcccaa gctgcatcat cgaaattgcc gtcaaccaag 12840

ctctgataga gttggtcaag accaatgcgg agcatatacg cccggagcct tggcgatcct 12900

gcaagctccg gatgcctccg ctcgaagtag cgcgtctgct gctccataca agccaaccac 12960

ggcctccaga agaagatgtt ggcgacctcg tattgggaat ccccgaacat cgcctcgctc 13020

cagtcaatga ccgctgttat gcggccattg tccgtcagga cattgttgga gccgaatccg 13080

cgtgcacgag tgccggactt cggggcagtc ctcgcccaaa gcatcagctc atcgagagcc 13140

tgcgcgacgg acgcactgac ggtgtcgtcc atcacagttt gccagtgata cacatgggga 13200

tcagcaatcg cgcatatgaa atcacgccat gtagtgtatt gaccgattcc ttgcggtccg 13260

aatgggccga acccgctcgt ctggctaaga tcggccgcag cgatagcatc catagcctcc 13320

gcgaccggct gaagaacagc gggcagttcg gtttcaggca ggtcttgcaa cgtgacaccc 13380

tgtgcacggc gggagatgca ataggtcagg ctctcgctga actccccaat gtcaagcact 13440

tccggaatcg ggagcgcggc cgatgcaaag tgccgataaa cataacgatc tttgtagaaa 13500

ccatcggcgc agctatttac ccgcaggaca tatccacgcc ctcctacatc gaagctgaaa 13560

gcacgagatt cttcgccctc cgagagctgc atcaggtcgg agacgctgtc gaacttttcg 13620

atcagaaact tctcgacaga cgtcgcggtg agttcaggct ttttcatagg ggggattcga 13680

gttgagagtg aatatgagac tctaattgga taccgagggg aatttatgga acgtcagtgg 13740

agcatttttg acaagaaata tttgctagct gatagtgacc ttaggcgact tttgaacgcg 13800

caataatggt ttctgacgta tgtgcttagc tcattaaact ccagaaaccc gcggctcagt 13860

ggctccttca acgttgcggt tctgtcagtt ccaaacgtaa aacggcttgt cccgcgtcat 13920

cggcggggtc ataacgtgac tcccttaatt ctcatgtatg ataattcgag ggtacccggg 13980

gatcct 13986

Supplementary Figure S1. Vector pH7GWIWG2(I) used for transformation of P. tremula x tremuloides.

Vector pH7GWIWG2(I)

LSD1

>gi|224115559|ref|XM_002317030.1| Populus trichocarpa predicted protein, mRNA (540 bp)

ATGCAGAGCCAGGTGGTGTGTAGAGGGTGTAGAAGTTTATTGTTATATCCAAGTGGAGCTACT

AATGTTTGTTGTGCTTTATGTAGTACCGTTACCTCTATTCCTGCTCCTGGGATGGACTTGGCTCA

ACTCATATGTCGAGGTTGCAGGACATTGCTAATGTATGCACGGGGGGCAACAACTGTGAGAT

GCTCCTGCTGCCACATAGTGAACTTTGCACCAGTAGGACCTAACCAGGTTGCTCATGTCAACT

GTGGGAACTGCCAGACTACACTTATGTATCCGAATGGTGCTCCATCTGTCAAGTGTGCGGTCT

GTCACTATGTTACTAACATTAGTACGGCTAATGTGAGAGTTCCGCTTCCAGCAAACAGACCTA

GTGGTATAGGTGGAACAATGCCGTCTACTTCAACAGTAAGTAACTGGACTTACTTTTCTTTCCT

GACTCAAACTGTCGTTGTGGAAAACCCTATGTCTGTTGATGAAAGTGGCAAATTGGTCAGCAA

TGTTGTTGTTGGTGTCACTACAGAAAAAAAAATAA (insert 86 bp)

EDS1

>gi|224121609|ref|XM_002318590.1| Populus trichocarpa disease resistance protein, mRNA (2084 bp)

GACGACGAAACCGACCATTTCTGCCTATAGTTTAACGCGTGTTTCATTCTTTCATGTCTGAAAG

ATAACAAGAAAGAATCCAAAGCTGCAAATTCCCGAAAAAATCGAAAGCCAGTGATGGGTAC

TGTCAAGCTTGGAGAGAACATGGAGATCAAAGAAGTGGTTATCCAGAAAGCATGCTCAATGG

CCATGAATGCTCACAAATCTCCAGAGAAGCAATATCTTTCTAAAAAGATTAGAACTTCATCAT

CTGAAGTTGTCTTTAGCTTTCCTGGATCTTGGTCTGTAAATGATTGGTTTGCTGGAACTTCCTTT

GGAGAAACGAAGATGGATCCTCAGCTCTTTCCTTCTCTCAAATATGTTGGTCTTGATGTAACTG

CTACTGTAAACGAAGTCTTTCTCAATAGATTCAAAGCAGTTTTGGCTAATCCACAGTTCCAAA

TTGAGGTGGAAAAAGCTGCGACAGACAGGAGGCAAATTGTATTTACAGGCCACTCTTCAGGA

GGGGCAATTGCCATACTGGCAACAATTTGGTTCTTGGAAGAGCAAATTAGAAAAAGCTCCAA

CTGGATTGCGCCACTCTGTCTGACTTTTGGATCTCCACTTGTTGGTGATCGCATTATAAACCTTG

CACTTAGGCGTGAAAACTGGTCTCGCTACTTTGTGAATTTCGTTATGAGATGCGACATTGTTCC

TCAGATTTCACTCAGTCCACTCTCTTCCATTAATCAAAAACTGCAGCAAGTTCTTGATTACTTT

AATCAAAAAGCGCAACAACCTCCTAATGAAGCTCCAGCTTTCTATGTAACTGTGGTGAAAAA

TGCATCGTCTGTTGCTAATTATGCTGCCTGTAAGATCATGGGAAGCACGAATCCTTTATTGGAG

ACTATATCAAGTTTCATTGAACTGAGTCCTTACAGACCCTTAGGGACCTATGTTTTCTGCACAG

GAAATGGGAAACTTGTTGTCTCAAGTAACCCAGATGCTGTGCTGCAAGTACTGTACTATGCCT

CCCAGTTGAGCACTGGAGAAGCAAGAGAAAAAGTTGAAGTTGCCCAAACAAGCCTAAGAGA

TCATTTGAACTACGGAAATGACTTGCAAGAATACTTAAAAATGTCAATTGTAACATGTTTATA

TCAGCATCATCCAGAAGCACTTCCGTTATCTTCGAATGTTGGAAATGTTGAGCGTGGGAGAGT

TGATGTGGCCTTGAATGACCTAGGCCTGAGTGAAAGAGCCAGACTGTGCATTCATGCTGCTGA

AGCGTTAGAGAAGCAGAAGTTGAGGAACCAGGCATCAATTGAAGAAAAGCAGAAAGATATT

GAGAAATGTTTAGATAAGCTAGAAGCGTACAAAAAGAAGGGTGCACTCAAAGTTGGCTATTA

TGATGCCTTCAAGAGTTCAGAACAAAAGGAGGATTTCCATGCTAATGTAGAGAGGCTTGAGC

TAGCAGGTATATGGGATGAAATAATAGAAATGCTGAAAAGGAATGAACTCCCCGATGAGTTT

GAGGGCCGGAAGACATGGATAGATCTTGGTACCAGGTATCGTCGCATTGTTGAACCCTTGGAT

ATTGCTAATTACTATCGACACCTCAAGAATGAAGACACAGGGCCCTATATGGGAAAGGGCAG

GCCAAGACGGTATAAATGCACCCAAAGATGGCGGGAGCATGCTGAGCGATTGCCGCATGAA

GTTCCAGGATCCTGTTTTTGGGCTGAGGTAGAGGAACTGTGCATTAAAACAAGCTGCCAGGGA

ATTAAAGAAAGCATTTCTCACCTAAATACCAAAGTCAAAAAGTGGATTAAAGATGGGGAACT

GGGTGTTGATGTTCTGTTGGAGAATTCCACCTTTAATAAATTGCTGAAACAACATTTCCTGACA

AACTTTTCTCAGGATCTTGACCTAAGGAAGGAGTTGCATGTGCAGGGATTTGCAGATGCGTAA

CTAATGTTATTCCGAGCCTTGTACACACCTGAGCAAGAACTTTATCTGTATTAGATATTTTATT

GTTATCTGTTCCACCCTGCAAAATAGTGTGAAATAAAATGCTTTTTTGATCAATAATAAAGGC

AGAAAGGGAA (insert 151 bp)

PAD4

>gi|224083495|ref|XM_002307015.1| Populus trichocarpa PAD4, mRNA (1509 bp)

ATGCAGACAAGCAAGTCAATTATCTTCACAGGCCATTCCGTAGGTGGAGCAACTGCCTCTCTT

GCTGCTCTTTGGCTCCTCTCTTACCTTCAATCAAATTTCTTAAACCTCTCGGTACTATGCATCAC

CTTTGGCTCTCCATTGCTAGGCAATGAGACCCTTTCTCGTGCCATTCTTCGCGAAAAATGGGGT

GGCAAATTTTGCCATGTTGTATCGAAGTATGACATCATGCCAAGGATGCTCTTTGTTCCAATGG

ATCCCATTGCTCCACTCTTAAAACCCCTGCTACATTTTTGGCACATGTATATGAATTCTCCGCA

TTTTGGATTGCTTGCTGTACCGTTAAGTGATGACAGCATGGCTCAAATTTTCCAGCATGTATTA

TTTCACTTGGGAAGATTAGTAGAAGCTGGAGAAGAGGCAGTGACTGGTGGAATGTTGAGGCC

ATTTGGGAACTATTTTTTCTGTTCGGAAGATGGAGCAATTTGCGTGGACAATGCAGCATCTGTT

GTTAAAATGATGTATTTGTTGTTCGCGACGGGGTTGCCTAGCTCTAGCATTGGGGATCATCTCA

AGTATGGTGATTACGTAGGGAAAATCTCCTTGCAATTTCTGGAGAAGAGGAGTTTCATGCAAG

GAGAGCTTCCTGAATCAAGCTACGAAGCAGGTGTTGTATTGGCTTTGCAGTCCACAGGAATAT

CTTGCAAGGAACAAATTGCAGGGCCAGCCAAAGATTGCCTAAAGGCTGCTAGGCGATTGGGG

CGCACACCAAACCTCAACTGTGCCAATCTGGCAATTAAACTGTCCAAAATCAATCCTTATAGG
GCTGAAATAGAGTGGTATAAAGCATTGTGCGACCGGTCTGATGATCAGATGGGTTACTACGA
CTCCTTTAAACAAAGGGGAGCCTCGAAAAGGGACTTCAAAGTCAACTTGAACCGGCACAAGC
TGGCTCAGTTTTGGGATAACGTAATAAACTTGTTTGAAAGCAATCAACTCCCCCATGATTTTCA
CAGACAAGGAAAATGGGTCAATGCTTCCCAGTTCTATAAGCTCCTTGTGGAGCCATTGGATAT
TGCTGAGTATTATCGAACAGGCATGCATCGTAGCAAGGGGCATTACATTGAACATGGAAGGG
AGAGGAGATATCGTATTTTTGATAGGTGGTGGAAAGAGAGAAGTGTTAGAGGGGAAAATTAT
AAACGGAGCAAGTTTGCAAGTTTGACCCAAGACACATGCTTCTGGGCAAGAGTGGAGGAAGC
CAGGGATTTGTTAGATGCTCTTAGAAGCACTAGTGATCCGAGTCATTTGGCTTTGCTTTGGCAG
AAGATAGATAGTTTTGCGAGCGATGCAAACGCGTTGGTTGAAACTAAGGAGGTGTCTATAGA
TGTAGTGGCAAAGAATTCAACCTATTCTTTGTGGCTAAAAGATTATAATGAATTGAAATCACA
AAAGGTGCAATTCCGTCCCTTGTTTTTGAGTTTTGTGAATGAGGAAATGGTTCCATAG (insert 79 bp)

Supplementary Figure S2. cDNA sequences of LSD1, EDS1 and PAD4. Short cDNA sequences inserted into silencing vector are highlighted in yellow, primers are highlighted in grey and underlined.

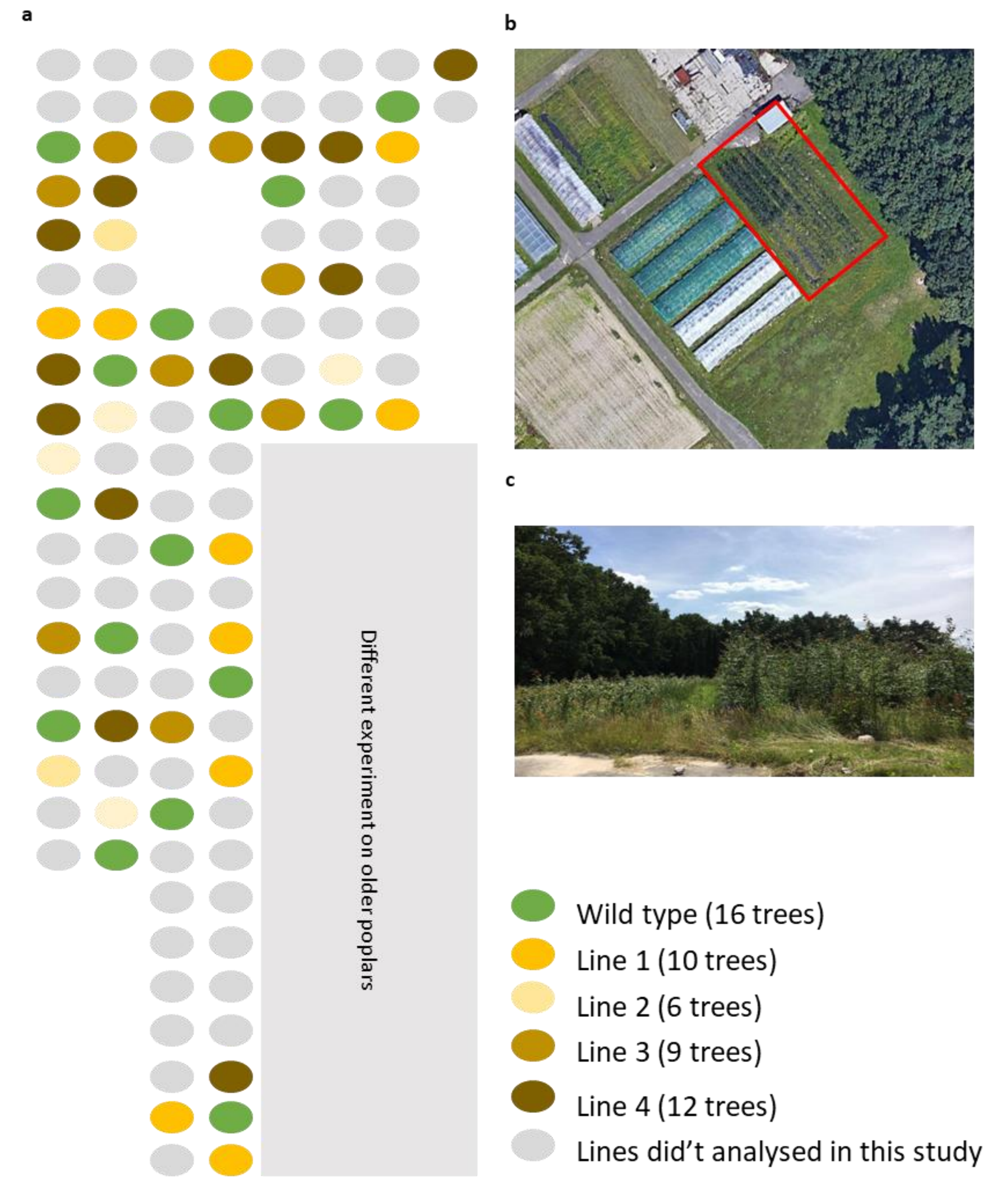

Supplementary Figure S3. Scheme of aspen cultivation and field experiment. Schematic presentation of experimental objects randomization in the field (a). Satellite image of the experimental field (GoogleMaps, USA), the field is marked with a red frame (b). Picture of the field experiment in the second year of the experiment (c).

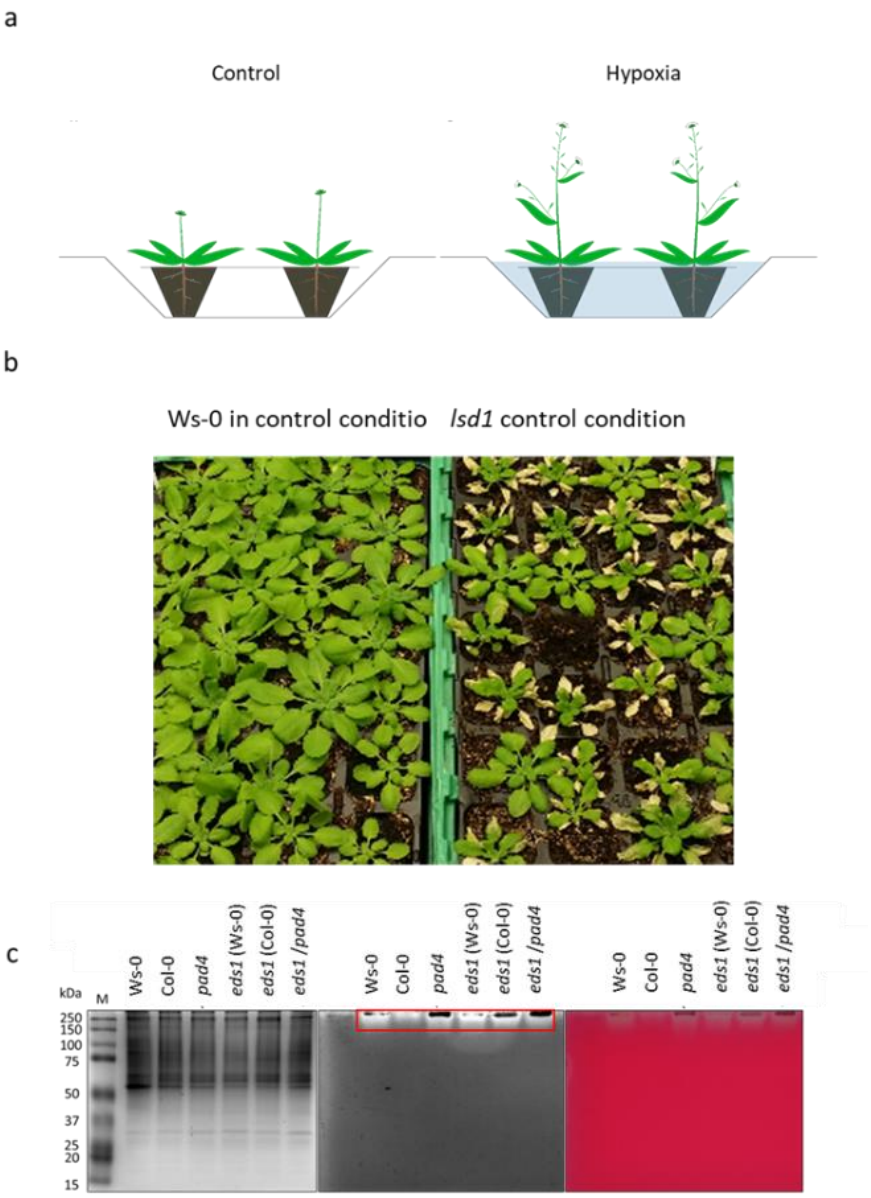

Supplementary Figure S4. Technical details of experiments. (a) Model of experimental setup for lysigenous aerenchyma formation in response to hypoxia stress. (b). Higher reducing sugar content in a lsd1 mutant before and after hypoxia stress was detected. It is easily explainable because the lsd1 mutant induces PCD during the 8-week growth in short photoperiod (8 hours of light and 16 hours of night) in controlled conditions and cannot survive the hypoxia stress. By contrast, a eds1 mutant increased its reducing sugar content during the hypoxia stress. We did not observe decrease in reducing sugar content during the hypoxia stress in any other genotype. Comparison of a 8-week-old Arabidopsis thaliana plant, Ws-0 (left) and lsd1 (right) (c). The eds1 and eds1/lsd1 mutants did not visually differ from Ws-0. Gel stained with Coomassie Brilliant Blue showing proteins of roots and

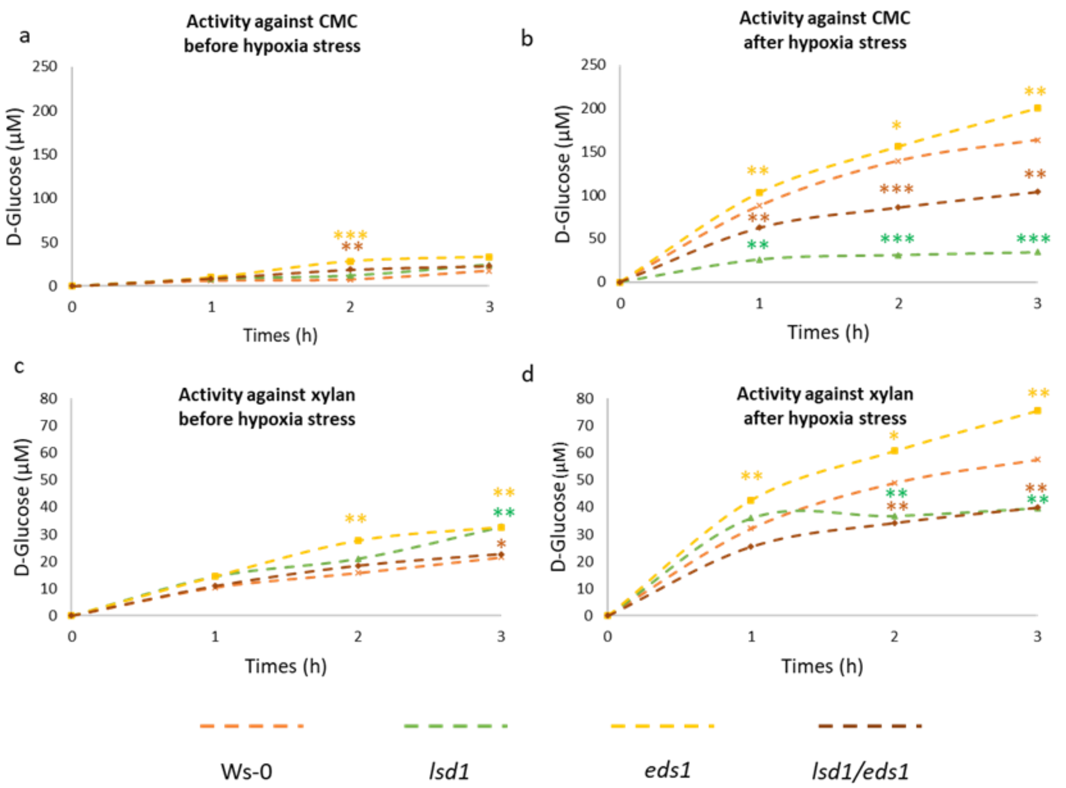

Supplementary Figure S5 Glucanase and xylanase activities in roots and hypocotyls measured after hypoxia stress. Protein extracts isolated before hypoxia stress exhibited lower activity against carboxymethyl cellulose (a) and xylan (c), compared to extracts isolated after hypoxia stress (b, d). Means values (±SD) are derived from separate biological replicates (n = 3 to 6). Stars above the bars indicate statistically significant differences in comparison to the WT plants, according to the Tukey HSD at level $p < 0.05$ (*), $p < 0.01$ (**), and $p < 0.001$ (***).

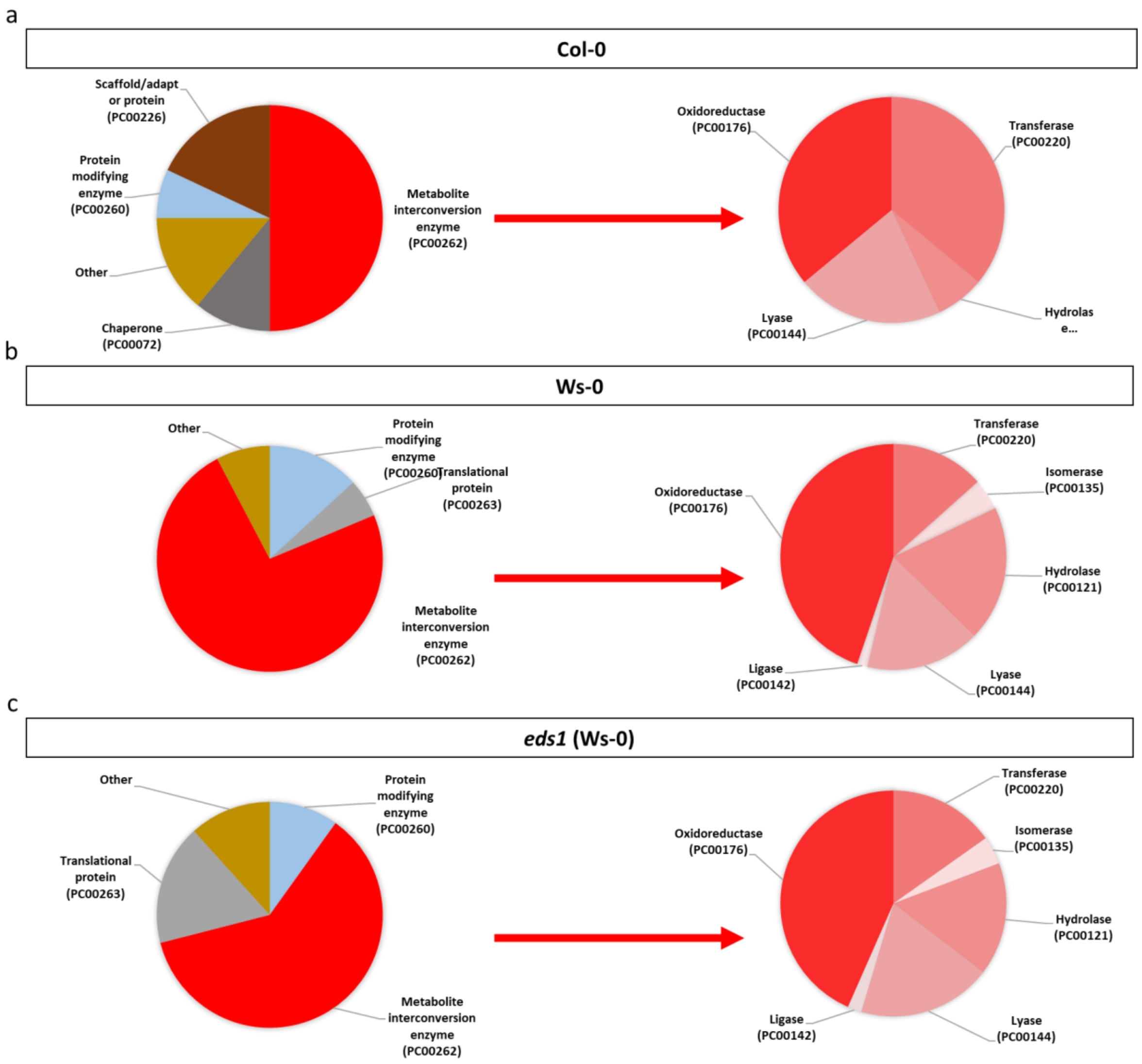

Supplementary Figure S6. Protein classes analysis by Panther.org. Pie chart presenting different protein classes taking part in hypoxia stress response and their abundance among identified proteins in tested genotypes; Col-0 (a), Ws-0 (b) and eds1 (Ws-0 background) (c) and pie chart presenting division of categories metabolite interconversion enzyme (PC00262) into subcategories (Right site). In order to obtain material for Maldi-TOF analysis, 6 separate experiments lasting one year in total were carried out. Then the Maldi-TOF analysis was performed and Data were based on 6 separate experiments (biological replicates) and two technical repetitions per sample. The results were analyzed using the Mascot software and trimmed so there would be not more than 1% of false positives in each sample.

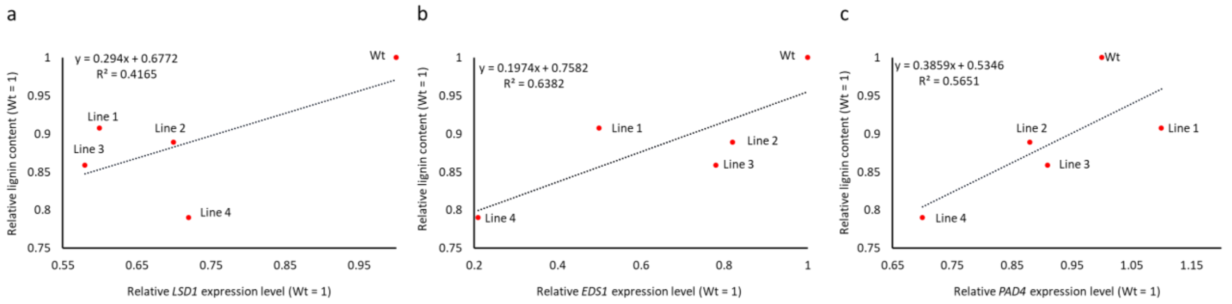

Supplementary Figure S7. Correlation of wood lignin content and deregulation of PtLSD1, PtEDS1 and PtPAD4 gene expression in leafs of tested lines. Correlation of lignin content (percent) and relative expression of LSD1 (a), EDS1 (b) and PAD4 (c).

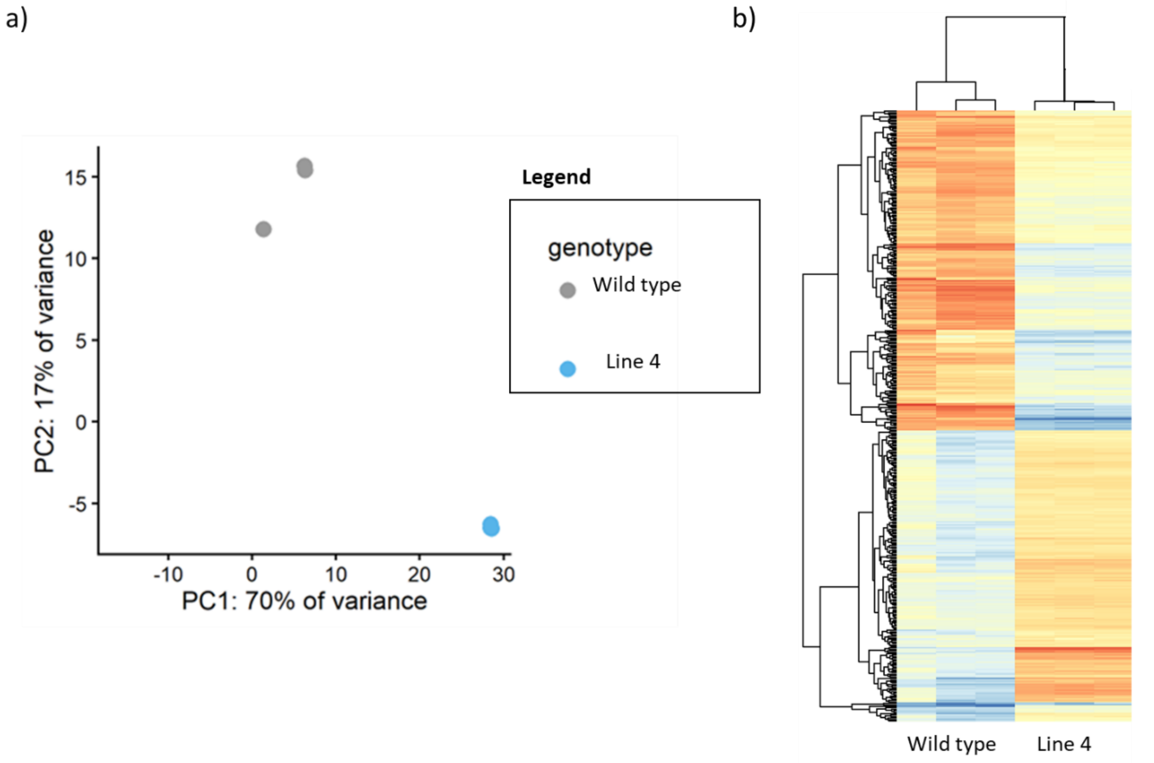

Supplementary Figure S8. RNAseq analysis of RNA isolated from cambium and differentiating xylem of wild type and transgenic Line 4 with the lowest relative lignin content (see Figure 3). The PCA analysis is showing huge differences between RNA samples isolated from cambium and differentiating xylem tissue of wood of wild type and Line 4 (a) and heat map of differently deregulated genes in wild type and Line 4 (b). The analysis was made on three independent biological repeats (n = 3).

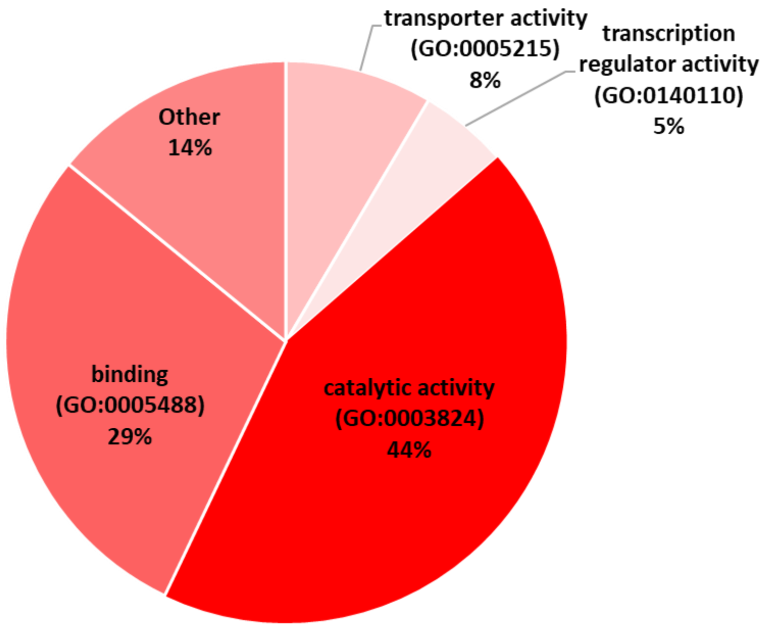

Supplementary Figure S9. Gene ontology analysis of deregulated genes in Line 4 in terms of their molecular function.

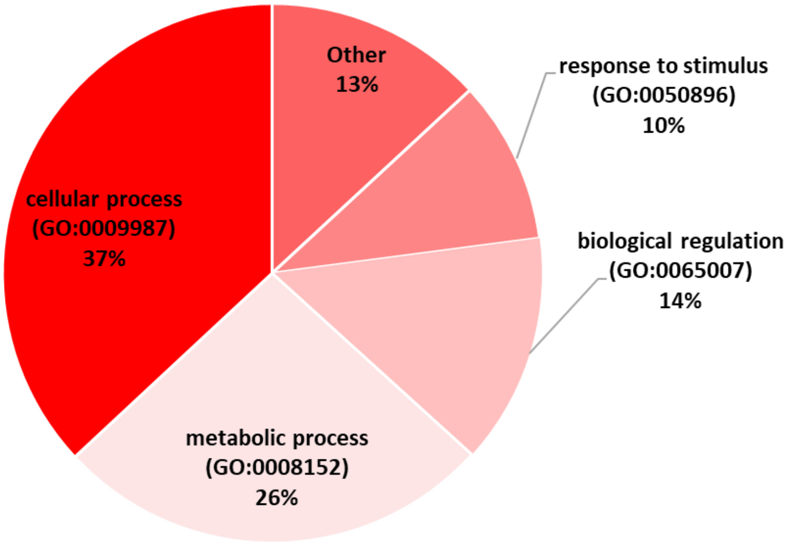

Supplementary Figure S10. Gene ontology analysis of deregulated genes in Line 4 in terms of biological processes.

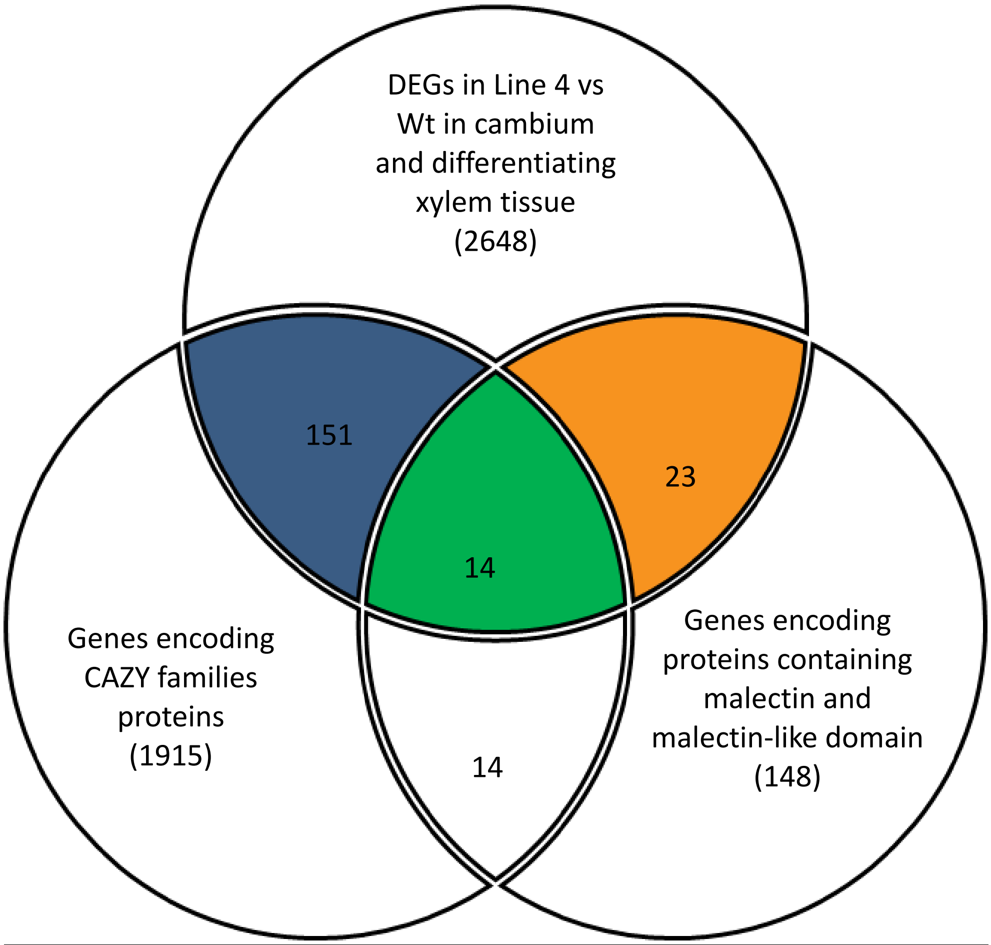

Supplementary Figure S11. Results of Line 4 DEGs and its comparison to genes encoding proteins from CAZY families and genes encoding malectin and malectin-like domain-containing proteins.

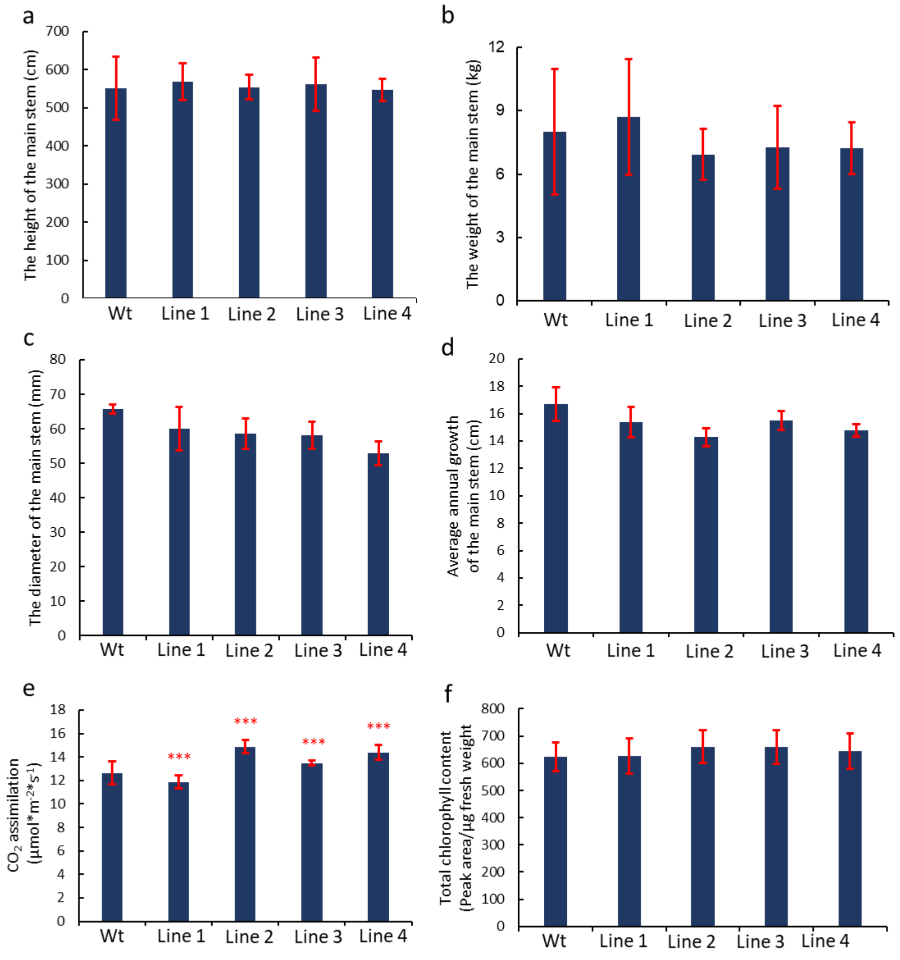

Supplementary Figure S12. Biometric parameters, CO_2 assimilation, and chlorophyll content in transgenic lines. Height of the main stem **(a)**, fresh weight of the main stem **(b)**, diameter of the main stem **(c)**, average annual growth of the main stem **(c)**, CO_2 assimilation **(d),** total chlorophyll content in leaves **(f)**. Stars above the bars indicate statistically significant differences in comparison to the WT plants, according to Tukey HSD at level $p < 0.05$ (*), $p < 0.01$ (**), and $p < 0.001$ (***). Mean values (±SD) for five to eleven different biological replications for data on graphs A and B (n = 5 to11), for three different biological replications on graph C (n=3), for four different biological replications on graph D (n = 4), for 30 different leaves *per* genotype on graph E (n = 30) and from nine different technical replications of pooled leaf sample *per* genotype on graph F (n = 9).
